# Detection and Quantification of Corn Starch and Wheat Flour as Adulterants in Milk Powder by Raman Spectroscopy Coupled with Chemometric Routines

**DOI:** 10.3390/s26041304

**Published:** 2026-02-18

**Authors:** Edwin R. Caballero-Agosto, Louang D. Cruz-Dorta, Samuel P. Hernandez-Rivera, Leonardo C. Pacheco-Londoño, Ricardo Infante-Castillo

**Affiliations:** 1 Department of Physics-Chemistry, University of Puerto Rico-Arecibo, Arecibo, PR 00614, USA; edwin.caballero@upr.edu (E.R.C.-A.); louang.cruz@upr.edu (L.D.C.-D.); 2 Center for Chemical Sensors (CCS), Chemical Imaging and Surface Analysis Center (CISAC), Department of Chemistry, University of Puerto Rico-Mayagüez, Mayagüez, PR 00681, USA; samuel.hernandez3@upr.edu (S.P.H.-R.); leonardo.pacheco@unisimon.edu.co (L.C.P.-L.); 3 Faculty of Basic and Biomedical Sciences-Barranquilla, Simon Bolivar University, Barranquilla 080002, Colombia

**Keywords:** Raman spectroscopy (RS), milk powder, adulteration, partial least squares (PLS), principal component analysis (PCA)

## Abstract

**Highlights:**

**What are the main findings?**

- Portable Raman spectroscopy has been shown to quantify adulterants in milk powder using chemometric routines.
- Results show errors of 0.76 and 0.77 %*w*/*w* for quantifying corn starch and wheat flour as adulterants, respectively, using partial least squares models.

**What are the implications of the main findings?**

- Study shows that portable Raman spectroscopy is an effective tool for quantifying adulterants in milk powder.
- The portable Raman modality can be used as a complementary in situ rapid screening technique by non-academic users to detect adulterants in milk and other foods.

**Abstract:**

Adulteration of milk powder (MP) is performed, especially in underdeveloped countries, by adding corn starch (CS) or wheat flour (WF) without mentioning it. Multiple techniques have been established to reduce these deceptive methods. Most of these techniques require samples to be sent to the laboratory for results through a time-consuming, expert-requiring, and destructive procedure. Raman spectroscopy (RS) has seen application due to the availability of portable modalities and its non-destructive, water-insensitive nature. Using principal component analysis (PCA), the differences and similarities between MP and the adulterants (CS and WF) have been evaluated. To quantify the percentages of CS and WF binary mixtures independently with MP, partial least squares regression (PLSR) has been employed. A total of 70 MP samples independently adulterated with CS and WF were prepared. Thirteen chemometric modes were developed by combining the first and second derivatives with Standard Normal Variate (SNV) and Multiplicative Scatter Correction (MSC) to quantify adulteration. The results obtained for CS and WF mixtures show errors of 0.76 and 0.77 %*w*/*w*, respectively, with the optimized math pretreatment. These results demonstrate that the portable RS modality can be used as an effective technique for detecting adulterants in milk powder.

## 1. Introduction

Food adulteration remains a persistent global challenge. It compromises food safety, economic fairness, and consumer confidence. Economically motivated adulteration is particularly problematic in powdered food commodities. In these commodities, visual inspection provides limited information about authenticity. As a result, rapid, reliable, and non-destructive analytical methods applicable throughout the food supply chain have become a research priority [1,2]. Milk powder (MP) is a critical case study. It has high commercial value, extensive international trade, and is frequently used in infant nutrition and processed foods.

Over the past five years, vibrational spectroscopy—including near-infrared (NIR), mid-infrared (MIR/FTIR), and Raman spectroscopy—has become a key tool for authenticating food and detecting adulteration [3,4,5,6]. These techniques provide molecular fingerprints tied to fundamental vibrational modes and enable rapid analysis with minimal sample preparation. When combined with chemometric and machine-learning methods, vibrational spectroscopy enables both qualitative classification and quantitative measurement of adulterants in complex food samples [7,8,9]. As a result, these methods are widely used for oils, spices, honey, meat, dairy, and powdered foods [10,11,12,13,14].

Milk powder adulteration often involves adding inexpensive bulking agents or nitrogen-rich compounds to inflate apparent mass or protein content. Infrared spectroscopy has played a key role in addressing this issue; recent studies show that FTIR and NIR spectroscopy, combined with multivariate analysis, can accurately detect and quantify adulteration—including starches, flours, melamine, and reconstituted milk solids [15,16,17,18]. However, debate persists regarding the robustness and transferability of targeted chemometric models when adulterant type, processing conditions, or natural compositional variability change. As a result, interest has grown in non-targeted and anomaly-detection approaches, which flag suspicious samples without prior knowledge of the adulterant [19,20].

Despite their analytical strengths, many infrared-based studies rely on benchtop laboratory instrumentation, creating centralized workflows that slow decision-making. This approach requires sample transport, specialized infrastructure, and trained staff—limitations that are especially critical in time-sensitive settings such as border inspection, warehouse screening, and on-site quality control. As a result, recent research emphasizes portable and handheld spectroscopic platforms that deliver actionable results directly at the point of need [21,22,23].

Portable Raman spectroscopy is particularly promising for analyzing powdered foods. It is non-destructive, relatively insensitive to water, and enables compact instrument designs. Recent studies demonstrate the feasibility of portable Raman systems for detecting food adulteration, including applications to milk powder and other dairy matrices [24,25,26]. Furthermore, surface-enhanced Raman scattering (SERS) has been explored to improve sensitivity to trace adulterants. However, challenges such as substrate reproducibility and field robustness remain active areas of discussion [27,28].

In this evolving research landscape, our group previously reported the detection and quantification of corn starch (CS) and wheat flour (WF) adulteration in milk powder using near- and mid-infrared spectroscopy with chemometric routines [29]. That work showed that infrared fingerprints, combined with optimized preprocessing and partial least squares regression (PLSR), could achieve low prediction errors for both adulterants. Building on this foundation, the present study extends the same analytical objective. We apply a portable Raman spectroscopy platform, providing a methodological continuation from benchtop infrared analysis to field-deployable vibrational sensing.

The primary aim of this work is to evaluate portable Raman spectroscopy, combined with chemometric routines, for detecting and quantifying CS and WF adulteration in milk powder. This study uses principal component analysis for exploratory assessment and partial least squares regression for quantification. Results show prediction errors below 1 %*w*/*w* for both adulterants. These findings highlight the promise of portable vibrational methodologies. Such methods can complement—and in some contexts replace—laboratory-based techniques, enabling faster decision-making and broader surveillance against food adulteration throughout the supply chain.

## 2. Materials and Methods

### 2.1. Samples

**Figure 1:**
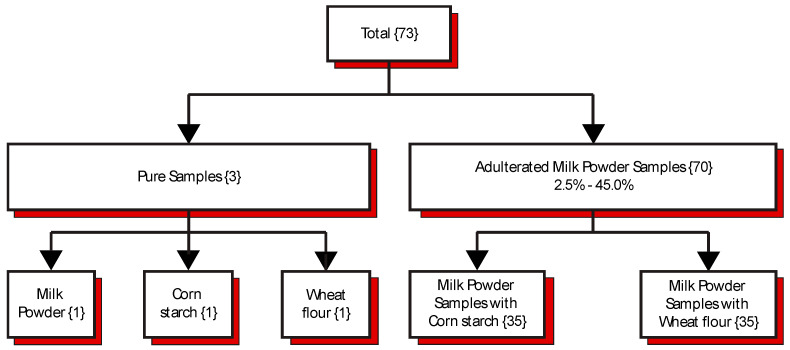
Sample preparation scheme (*n* = 73). Three samples of pure compounds (1 sample of milk powder, 1 sample of corn starch, and 1 sample of wheat flour), 35 samples of milk powder adulterated with corn starch, and 35 samples of milk powder adulterated with wheat flour.

This study used Denia^®^ whole milk powder (distributed by V. Suarez & Co., Bayamon, PR, USA), containing 26% milk fat from pasteurized cow’s milk. The whole milk powder (MP) contains palmitate, vitamin D3, calcium, riboflavin, and phosphorus. The corn starch (CS) used is Maizena^®^ (distributed by Unilever, Englewood Cliffs, NJ, USA; product of Mexico), which contains sulfur dioxide as a preservative. The wheat flour (WF) used is Enriched Wheat Flour (distributed by Goya Foods, Inc., Jersey City, NJ, USA), containing niacin, iron, thiamine, riboflavin, and folic acid. These specific brands were used due to their affordability and market accessibility, as well as their physical properties similar to those of the adulteration target. Figure 1 shows a schematic of the samples used in this study. From this scheme, one pure sample of MP, two pure samples of adulterants (CS and WF), and 70 different binary mixtures were prepared in this study.

In Table 1, adulterated samples were prepared by adding varying amounts of adulterants (CS and WF) to milk powder. The procedure was performed as follows: each sample of milk powder was manually mixed with the adulterant powder in a test tube, then placed on a high-speed stirrer with a glass rod to reduce particle-size variation. Samples were prepared by varying the proportions of milk powder and adulterant, and the range of percentage of adulterant, in this case, is between 2.5 and 45 %*w*/*w* adulteration. A total of 35 binary samples were mixed using CS and MP, and another 35 binary samples were mixed for CS and WF in the same manner.

### 2.2. Raman Spectroscopy Analysis

The milk powder mixtures were analyzed without pretreatment at room temperature (25 °C). The surface of the mixture was scanned using a handheld Raman spectrometer, Progeny^TM^ (Rigaku Analytical Devices, Wilmington, MA, USA). The spectrometer uses a 1064-nm laser and thermoelectrically cooled InGaAs 512-pixel detector with a spectral range of 145–2500 cm^−1^ at 8 cm^−1^ spectral resolution. The selection of the 1064 nm excitation wavelength was guided by the need to minimize fluorescence interference and ensure stable, reproducible Raman spectra for complex organic and powdered food matrices. Shorter excitation wavelengths, such as 532 nm and 785 nm, provide higher Raman scattering efficiency but are more susceptible to fluorescence background, which can obscure Raman features and adversely affect quantitative analysis in samples containing fluorescent groups [30,31]. In contrast, near-infrared excitation at 1064 nm operates at lower photon energy, significantly reducing fluorescence contributions and improving baseline stability, although with reduced scattering intensity [32]. This trade-off is particularly advantageous for organic materials, where fluorescence suppression is critical for reliable multivariate analysis and robust chemometric model development. Consequently, the use of a 1064 nm laser in this study improved spectral quality and reproducibility, supporting accurate, transferable quantitative modeling for routine screening applications. The laser power was set at 490 mW, and the exposure time was 30 s, with three averages to maximize the signal-to-noise ratio without damaging the sample. Six spectra were collected for each sample from six different points on the surface of the mixture. The optical setup used was backscattering, with the handheld spectrometer placed perpendicular to the table and the surface of the mixture to acquire the sample.

### 2.3. Spectral Data Processing

The collected Raman spectra were transformed into a data matrix with predictor (*X*) and response (*Y*) variables: Raman spectra (*X*) comprising Raman shifts as variables and samples as objects, and adulterant weight percentages (*Y*). Chemometric models were developed using The Unscrambler X^TM^ Ver. 10.5.1 software (The Unscrambler X, CAMO Analytics AS, Oslo, Norway). Principal component analysis (PCA) was used to identify the presence of adulterants in MP and detect outliers, while partial least squares regression (PLSR) was used to quantify the weight percentage of adulterants in MP.

All the developed models were optimized using a random cross-validation method included in the software package, with 20 segments, the NIPALS algorithm, and seven latent variables (principal components for PCA and factors for PLSR). The optimal number of latent variables was kept to 1 to ensure the model is as parsimonious as possible while minimizing under- and overfitting. To determine the mathematical treatment that effectively reduces spectral artifacts, twelve different combinations of pre-treatments were used, comprising Standard Normal Variate (SNV) and Multiplicative Scatter Correction (MSC) for correcting scattering and first (SG1) and second (SG2) order derivatives using Savitzky–Golay’s algorithm with a fixed polynomial order of 2 and 15 smoothing points. These combinations yield the following 12 mathematical treatments: MSC, SNV, SG1, SG2, MSC + SG1, MSC + SG2, SNV + SG1, SNV + SG2, SG1 + MSC, SG2 + MSC, SG1 + SNV, and SG2 + SNV. Including unprocessed spectral data (RAW), a total of 13 PLSR models were developed per adulterant, yielding 26 PLSR models overall.

The best mathematical pre-treatments were selected based on the statistical criteria for each adulterant. This selection was based on the lowest Root Mean Square Error of Prediction (RMSEP) and Relative Standard Error of Prediction (RSEP), as well as the highest Ratio of Prediction (RPD) and Range Error Ratio (RER). The 35 adulterant mixtures were divided into two sets: one for developing the PLSR model (CAL) and one for external testing of the developed model (TEST). The mixtures were divided using the 30/70 TEST/CAL split, ensuring that the TEST samples are between CAL samples and that the distribution is as symmetric as possible to ensure interpolation-based prediction while minimizing bias and extrapolation. This split can be viewed as a systematic, interleaved, space-filling external validation sampling procedure, with the CAL set at 70% (*n* = 26 samples) and the TEST set at 30% (*n* = 9 samples). External validation was performed using a systematic, interleaved, space-filling sampling strategy, chosen to ensure an unbiased and representative assessment of model performance. This design ensured that the TEST set spanned the full adulteration range (avoiding bias from concentration clustering) while remaining independent of calibration. For each binary system (MP–CS and MP–WF), the complete concentration series was first ordered from lowest to highest adulterant level. Samples were then assigned in an interleaved manner: approximately every third concentration was selected for the TEST set, while the remaining samples were used for calibration. This resulted in a 30/70 TEST/CAL split (9 TEST and 26 CAL samples). This procedure ensured that TEST samples were evenly distributed across the concentration range and fell within calibration levels, thereby enabling model evaluation via interpolation rather than extrapolation. The specific concentration values assigned to the CAL and TEST sets for both CS and WF are summarized in Figure S1 and Table S1 (Supplementary Materials), which visually represents the space-filling validation design.

## 3. Results and Discussion

**Figure 2:**
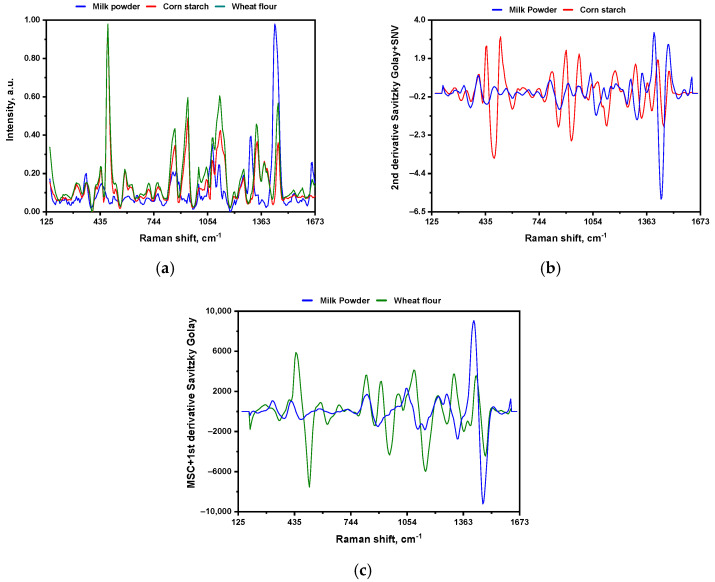
2D line plot of milk powder: (**a**) raw Raman spectra overlapped with corn starch (CS) and wheat flour (WF), (**b**) pre-treated spectra overlapped with CS, and (**c**) pre-treated spectra overlapped with WF. The mathematical pretreatments are the same as those used for the optimal PLSR models (SG2 + SNV for CS and MSC + SG1 for WF).

All samples were scanned with the previously detailed acquisition settings and optical setup for the portable Raman spectrometer. Figure 2 shows the raw average spectra of pure MP, CS, and WF.

### 3.1. Spectral Analysis

Figure 2 shows the FT-Raman spectra of milk powder (blue), corn starch (red), and wheat flour (green). A clear qualitative distinction among the samples is evident, primarily attributable to variations in the contributions of lipids, proteins, and carbohydrates. The spectral differences across the samples provide an initial indication of compositional variability, which is fundamental for subsequent chemometric modeling.

The main vibrational bands of milk powder, along with their assignments from previous reports [33,34], are listed in Table 2. The most distinctive feature appears at 1747 cm^−1^, corresponding to the ester carbonyl stretching vibration (*ν*) of triglycerides in milk fat. This band is intense in pure milk powder and markedly reduced in samples with starch or flour additives, confirming its diagnostic value for lipid content.

Another important region, at 1656 cm^−1^, comes from amide carbonyl stretching (*ν*) vibrations tied to milk proteins. This band is clearly visible in milk powder, but its intensity decreases in the adulterated sample, indicating dilution of the protein upon addition of starch or flour. Protein-related information also comes from the 1002 cm^−1^ band, assigned to phenylalanine ring-breathing vibrations, a stable aromatic marker in protein-rich matrices.

The region between 1380 and 1450 cm^−1^, particularly the band at 1440 cm^−1^, is associated with C–H deformation vibrations (*δ*) of methyl and methylene groups from fatty acid chains. This band shows higher intensity in the milk powder spectrum and progressively decreases in wheat flour and corn starch, further confirming its association with lipid content and its usefulness as a qualitative indicator of milk fat dilution.

Bands at 1262 cm^−1^ and 1303 cm^−1^ are associated with mixed organic matrix vibrations, predominantly deformation modes (*δ*) involving C–H and C–O groups. These bands reflect contributions from proteins, lipids, and carbohydrates and vary with sample composition, reinforcing their role as secondary indicators of adulteration.

The carbohydrate-dominated spectral region (920–1160 cm^−1^) shows pronounced overlap among milk powder, corn starch, and wheat flour. In milk powder, this region is linked to lactose, the main sugar in milk, with strong C–O (carbon-oxygen) and C–C (carbon-carbon) stretching vibrations (*ν*), referring to the movement of atoms during bond stretching. The 1079 cm^−1^ and 1123 cm^−1^ bands, associated with glycosidic (sugar bond) and glucosyl (glucose unit) stretching modes, are also strong in milk powder. Because starch and flour have polysaccharide (complex carbohydrate) structures, they exhibit similar features, leading to noticeable spectral overlap in mixtures. As a result, this region is highly sensitive to adulteration, and reliable discrimination still requires multivariate analysis, a statistical method that uses multiple variables to distinguish between samples.

The 872 cm^−1^ band is attributed to deformation-type vibrations (*δ*) tied to carbohydrate skeletal modes. The 355 cm^−1^ band is characteristic of lactose and confirms the presence of milk-derived components. These lower-intensity, lower-wavenumber bands help define the fingerprint region and together enhance qualitative interpretation.

Qualitative FT-Raman spectral analysis shows that lipid (fat) and protein-specific (amino acid-based) spectral bands at 1747, 1656, 1440, and 1002 cm^−1^ are highly specific for identifying milk powder. In contrast, the carbohydrate-rich region (920–1160 cm^−1^), reflecting sugars and starches, is most affected by corn starch and wheat flour. As shown in Figure 2, these compositional differences are clearly visible in the Raman profiles, thereby supporting chemometric (statistical) classification and adulteration detection.

In addition to qualitative band assignments, the Raman spectra exhibited systematic, concentration-dependent intensity changes as CS and WF levels increased. Specifically, bands within carbohydrate-related regions (323–380 cm^−1^, 454–540 cm^−1^, and 1420–1500 cm^−1^) showed consistent intensity trends with adulterant concentration. This indicates their relevance for quantitative analysis. Individual peak intensity ratios can be sensitive to local heterogeneity, particle size, and baseline variability in powdered samples. Instead of relying on these ratios, we used multivariate analysis to capture correlated variations in intensity across the full spectral range. This confirms that the dominant spectral variation captured by PCA is strongly associated with adulterant concentration.

In Raman analysis of carbohydrate-rich mixtures, pronounced overlap between the 920–1160 cm^−1^ C–O and C–C vibrational modes of lactose and polysaccharides complicates spectral interpretation. To clarify the rationale for each preprocessing method, SNV and MSC are applied to normalize intensities and correct for scatter, specifically minimizing baseline differences to allow accurate comparison [35,36]. Savitzky–Golay (SG) derivatives enhance resolution by smoothing noise and highlighting inflection points and peak curvature, targeting the separation of overlapping carbohydrate features [37]. Ruffin et al. generated synthetic spectra composed of multiple overlapping Gaussian bands and showed that Savitzky–Golay derivatives accurately detect each band [38]. These tailored methods—SG2 + SNV for corn starch (CS) and MSC + SG1 for wheat flour (WF)—were selected to enhance discrimination in the 920–1160 cm^−1^ region, enabling more apparent PCA-based sample separation and more robust, stable PLS calibration models.

### 3.2. Principal Component Analysis (PCA)

**Figure 3:**
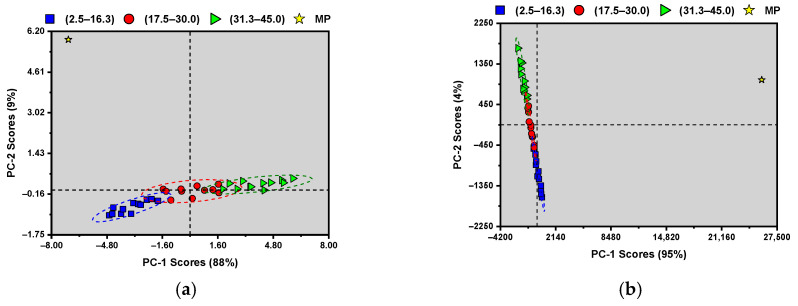
Principal component analysis of (yellow star) milk powder and their corresponding mixtures: (**a**) corn starch (CS) mixtures and (**b**) wheat flour (WF) mixtures. Mixtures were divided into three regions: (blue squares) 2.5–16.3, (red circles) 17.5–30.0, and (green triangles) 31.3–45.0 %*w*/*w*. A confidence ellipse with a 0.05 significance level was determined for each mixture cluster.

After plotting the spectra, PCA was performed using the pure MP spectra and the corresponding adulterant mixtures. For the development of the PCA and PLSR models, two independent binary systems are being studied (MP-CS and MP-WF). The resulting PCA and PLS models cannot simultaneously discriminate or quantify both adulterants. After determining the optimal math pre-treatment for the PLSR models, PCA models were also developed utilizing the optimal pre-treatments. Figure 3 summarizes the best-obtained results.

Figure 3 shows that it is possible to identify MP separated from the rest of the samples for both CS (a) and WF (b). The adulterant samples can be discriminated into three predetermined concentration regions, with well-defined, separated clusters. The CS mixtures (Figure 3a) appear to increase in concentration from bottom to top and left to right, with the middle range centered at the origin of the scores plot. Similarly, WF mixtures show a middle range centered on the origin, with samples increasing in concentration from bottom to top and right to left. For both scores, the pure MP sample aligns with the direction of the cluster with the least adulterant. For CS, a slight separation is observed between the pure MP mixture and the sample with the lowest adulterant level in the first component.

In contrast, a significantly larger gap is observed in the second component. For WF, the same ample space is observed; however, in the component and the second components, the MP sample is placed within the range with the highest adulterant concentration. The behavior shown in both score plots indicates that the first component separates the adulterant mixtures and the pure MP sample.

To verify mixture homogeneity, given the localized sampling nature of Raman spectroscopy, the six replicate spectra collected from six different positions within each prepared sample were used to quantify within-sample variability. For representative mixtures across the tested concentration range, peak intensities (or integrated band areas) were extracted from three prominent Raman regions (323–380 cm^−1^, 454–540 cm^−1^, and 1420–1500 cm^−1^), and the relative standard deviation (RSD, %) across the six spatial replicates was calculated for each region. An overall homogeneity indicator was then obtained by averaging the RSD values across the selected bands and concentrations for each binary system. Using this approach, the mixtures exhibited overall RSD values of no more than 12%, indicating that the manual mixing and high-speed stirring procedures produced sufficiently homogeneous samples for subsequent chemometric modeling.

### 3.3. Partial Least Squares Regression (PLSR) Results

This study evaluated two binary adulteration systems: milk powder–corn starch (MP–CS) and milk powder–wheat flour (MP–WF). The aim was not to compare corn starch and wheat flour but to test whether portable Raman spectroscopy and chemometric modeling could detect and quantify each adulterant separately in milk powder. For this reason, the models consider only binary adulteration, not cases with multiple adulterants or simultaneous identification of different adulterants. Distinguishing between CS and WF was outside the scope of this study.

After evaluating the classification procedure using raw spectra, the next step was to develop calibration models using all spectra with PLSR and cross-validation using random groups. As indicated in Section 2, different mathematical pre-treatments will be used to generate a total of thirteen PLSR models per adulterant. Table 3 summarizes the results obtained for each treatment and adulterant.

Although using a single latent variable (*LV* = 1) in PLSR models may seem unusual, this choice is justified in the present study. The application involves highly collinear Raman spectral data, binary adulteration systems, and a small sample size. Additional latent variables were tested during model optimization (Tables S3 and S4 in the Supplementary Materials). Higher LVs yielded only marginal calibration improvements (no more than 30% with 5 LVs) and did not consistently improve external validation. With an interleaved external validation scheme and moderate sample size (*n* = 70), retaining only the first latent variable minimized overfitting and maintained predictive stability. Explained variance analysis showed that the first LV accounted for over 70% of both X- and Y-variance, confirming that it captured the main chemical information relevant to adulterant concentration without underfitting. This streamlined strategy prioritizes robustness in real-world spectral acquisitions by buffering against the effects of added spectral artifacts (baseline, scattering, noise, etc.).

When analyzing the optimized PCA models (Figure 3), the first principal component (PC1) explains 88% and 95% of the total X-variance for the CS and WF systems, respectively. PC2 explains an additional 9% for CS and 4% for WF, resulting in cumulative variances of 97% and 99%. The PCA score plots show separation of the concentration levels along both PC1 and PC2. It is important to note that PCA is an unsupervised method that maximizes the variance of the spectral data (*X*) without considering the response variable (*Y*). As a result, variance captured by PC2 may reflect orthogonal spectral variability—such as scattering effects, baseline variation, or sample heterogeneity. These factors aid visual clustering but do not necessarily contribute to predicting adulterant concentration. Figure 3b appears to show PC2 wider than PC1; however, PC2 spans from −2250 to 2250, while PC1 spans from −4200 to 2140. Tables S3 and S4 (Supplementary Materials) present the optimal LVs per PLSR model for CS-MP and WF-MP, along with a discussion of why model simplicity was favored over predictive power for both PLSR models. In contrast, PLSR LVs are extracted to maximize the covariance between X and Y, ensuring that retained components are directly relevant to concentration prediction. Consistent with this distinction, evaluating the X-variance and Y-variance explained versus LVs showed that the first LV alone captured more than 70% of the relevant information for both CS and WF. Additional LVs did not consistently improve external validation performance (Tables S3 and S4, Supplementary Materials). Given the limited dataset size and the interleaved external validation design, retaining a single latent variable may help reduce the expected decrease in robustness when using a portable Raman spectrometer outside a controlled environment by non-academic personnel. This approach minimizes overfitting while preserving predictive stability.

As can be seen in Table 3, for CS, all RMSEP values are below 6.56 %*w*/*w* with the median being 0.81 %*w*/*w*, RSEP are below 26.0% with the median being 3.2%, RPD are above 1.8 with the median being 11.1, and RER are above 5.8 with the median being 31.4. For WF, all RMSEP values are below 3.54 %*w*/*w*, with the median being 0.92 %*w*/*w*; RSEP values are below 14.0%, with the median being 3.6%; RPD values are above 2.5, with the median being 9.5; and RER values are above 6.5, with the median being 29.5. The lowest performance can be seen for the RAW PLSR models, while the best mathematical pre-treatment for adulterants was SG2 + SNV for CS and MSC + SG1 for WF. Table 4 summarizes the optimal PLSR model determined for both adulterants.

**Figure 4:**
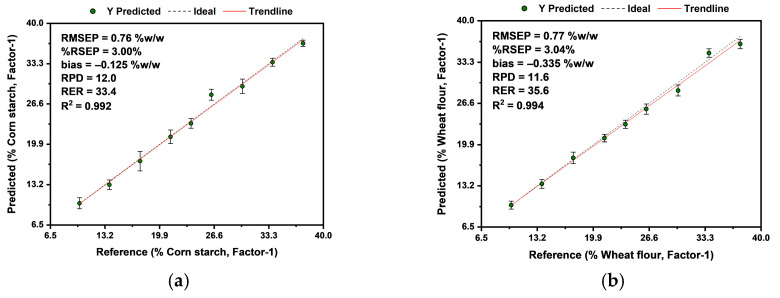
Predicted vs. reference plot showing the performance of the optimal PLSR model for (**a**) corn starch and (**b**) wheat flour. The models with samples and red line (model linear fit) close to the black dashed lines (ideal model) illustrate higher accuracy, while the shorter whiskers indicate higher precision.

As shown in Table 4, the optimal PLSR test models had an average of 0.77 %*w*/*w* RMSEP, 3.0% RSEP, 11.8 RPD, and 34.5 RER for adulterants, with CS having the best-performing model by a slight margin. The calibration and cross-validation results were also shown in Table 4, with the test models showing surprisingly better performance in RMSEP and RSEP; however, the optimistic results of calibration and cross-validation appear in the RPD and RER values. As mentioned in Section 2.3, the number of factors used was 1 for all the models. After analyzing the PCA scores plot with the optimized mathematical pre-treatments in Figure 3, further evidence is provided for using a single latent variable as a reasonable selection. Figure 4 shows the predicted vs. reference plot for the optimal PLSR models for both adulterants.

The primary objective of this study was not trace-level detection. Instead, it aimed for reliable discrimination and quantification of economically meaningful adulteration levels in milk powder. In practical food fraud scenarios, adulterants such as CS and WF are introduced at concentrations well above trace levels. Very low percentages do not provide enough economic incentive, given the risk and processing effort [39]. The selected concentration range reflects realistic adulteration practices seen in routine quality control and screening. It does not represent the detection limits of the spectroscopic technique. Therefore, performance metrics such as prediction error (RMSEP) and model stability under external validation were considered more suitable.

Portable Raman spectroscopy offers key advantages for point-of-need analysis, including rapid measurements, minimal sample preparation, and in situ use. These features support this study’s aims and make portable systems suitable for food authentication and field screening. However, portable Raman instruments face operational limitations compared with laboratory benchtop systems: lower laser power, reduced optical throughput, and decreased detector sensitivity (due to eye-safety regulations, battery limitations, and compact design) can degrade signal-to-noise ratio and spectral resolution in complex samples [40]. Also, fluorescence interference is often higher in portable systems because of limited excitation wavelength options and simpler optics [41]. By contrast, benchtop Raman systems offer higher laser power, better detector cooling, and flexible data acquisition, resulting in improved fluorescence suppression and spectral resolution. Thus, portable Raman is a complementary tool for rapid screening when combined with robust chemometric analysis, as shown in this study.

Previous near- and mid-infrared (NIR/MIR) spectroscopy studies, combined with chemometric modeling, have demonstrated effective detection and quantification of CS and WF adulteration in MP under laboratory conditions. For example, optimized FT-NIR and FT-MIR models typically achieve prediction errors below or near 1 %*w*/*w* when benchtop instruments and controlled sampling geometries are used [29]. However, these infrared approaches generally require contact-based or ATR configurations and stable environmental conditions, which may limit their use for rapid, on-site analysis. In contrast, the present work shows that portable Raman spectroscopy can achieve similar prediction accuracy for individual binary adulteration systems. Furthermore, it offers practical advantages, such as non-contact measurements, reduced sensitivity to water absorption, and simplified sample handling. Taken together, these results position portable Raman spectroscopy as a complementary, field-deployable alternative to established NIR/MIR methods, rather than a replacement.

Recent literature shows that machine-learning and deep-learning methods can improve Raman spectroscopy, especially for complex or large-scale datasets, by automating preprocessing and delivering higher accuracy in certain classification and regression tasks [42]. Despite these benefits, reviews note key limitations, such as high computational cost, large data requirements, limited interpretability, and greater complexity. For studies with moderate data that require transparent and easily deployable models, classical chemometric methods such as PCA and PLS continue to offer direct advantages, including robustness, interpretability, compatibility with standard analysis software, accessibility, and reproducibility. In this work, PCA and PLS were selected to ensure these advantages, in line with recent conclusions that deep learning is justified only when a problem’s complexity and data clearly outweigh the strengths of existing methods [40].

## 4. Conclusions

In this work, we determined whether a portable Raman spectrometer coupled with chemometric routines (PCA and PLSR) could be used for fraud detection in milk powder. Detecting fraud in solid samples, such as milk powder, requires the collection of robust spectra that provide relevant information. All classification and calibration models showed satisfactory results. The optimal PLSR model for quantifying CS was developed using SG2 + SNV math pre-treatment combination, while WF was developed using MSC + SG1. For all adulterants, the RMSEP is 0.77 %*w*/*w* on average, with an RSEP of 3.0%. These results confirm that portable Raman spectrometers can be used to detect food fraud in milk powder samples and open the possibility of detecting other solid mixtures in the food industry, as well as malicious adulteration of solid samples in other relevant fields.

## Figures and Tables

**Table 1:** Percentage distribution of binary (milk powder and one adulterant) mixtures.

Range	Component	Weight-Weight Percentage (%*w*/*w*)											
Low	Milk Powder	97.5	96.3	95.0	93.7	92.5	91.2	89.9	88.7	87.5	86.2	85.0	83.7
	Adulterant	2.5	3.7	5.0	6.3	7.5	8.8	10.1	11.3	12.5	13.8	15.0	16.3
Medium	Milk Powder	82.5	81.3	80.0	78.7	77.5	76.2	75.0	73.7	72.5	71.3	70.0	-
	Adulterant	17.5	17.7	20.0	21.3	22.5	23.8	25.0	26.3	27.5	28.7	30.0	-
High	Milk Powder	68.7	67.5	66.3	65.0	63.8	62.5	61.3	60.0	58.8	57.5	56.2	55.0
	Adulterant	31.3	32.5	33.7	35.0	36.2	37.5	38.7	40.0	41.2	42.5	43.8	45.0

**Table 2:** Main Raman shifts in cm^−1^, and their respective tentative assignments for milk powder.

Raman Shift (cm^−1^)	Band Strength	Assignment
355	weak	Lactose
442	weak	$\delta \;\left(C-C-C\right)+\tau \;\left(C-O\right)$
872	weak	$\delta \;\left(C-C-H\right)+\delta \;\left(C-O-C\right)$
1002	weak	Ring-breathing (phenylalanine)
1079	medium	$\nu \;\left(C-O\right)+\nu \;\left(C-C\right)+\delta \;\left(C-O-H\right)$
1123	weak	$\nu \;\left(C-O\right)+\nu \;\left(C-C\right)+\delta \;\left(C-O-H\right)$
1262	weak	$\gamma \;\left(CH_{2}\right)$
1303	medium	$\tau \;\left(CH_{2}\right)$
1440	very strong	$\delta \;\left(CH_{2}\right)$
1656	medium	$\nu \;\left(C=O\right)Amide;\nu \;\left(C=C\right)$
1747	weak	$\nu \;\left(C=O\right)$Ester

ν—stretcing vibrations, δ—scissoring (symmetric deformation) vibrations, τ—twisting, γ—out-of-plane deformation.

**Table 3:** Test statistics of developed partial least squares regression (PLSR) models to quantify adulteration of milk powder with corn starch and wheat flour (*n* = 70).

Adulterant	PLS Parameters	RAW	MSC	SNV	SG1	SG2	MSC + SG1	MSC + SG2	SNV + SG1	SNV + SG2	SG1 + MSC	SG2 + MSC	SG1 + SNV	SG2 + SNV
Corn starch	RMSEP [%*w*/*w*]	6.56	0.96	0.98	2.01	1.96	0.79	0.76	0.8	0.76	0.83	0.76	0.81	0.76
	RSEP [%]	26.0	3.8	3.9	7.9	7.7	3.1	3.0	3.2	3.0	3.3	3.0	3.2	3.0
	RPD	1.8	9.6	9.5	5.1	5.2	11.2	11.9	11.2	12.0	10.6	11.8	11.1	12.1
	RER	5.8	26.1	25.7	14.7	15.2	31.4	33.0	31.7	33.1	31.3	33.1	32.5	33.6
Wheat flour	RMSEP [%*w*/*w*]	3.54	0.83	0.83	1.10	0.92	0.77	0.80	0.84	0.86	1.13	1.18	1.12	1.16
	RSEP [%]	14.0	3.3	3.3	4.4	3.6	3.0	3.2	3.3	3.4	4.5	4.6	4.4	4.6
	RPD	2.5	11.0	11.1	7.7	9.5	11.6	11.3	10.7	10.6	7.9	7.7	8.0	7.8
	RER	6.5	33.6	33.7	22.9	28.5	35.6	34.5	35.0	33.6	28.9	27.4	29.5	27.6

RAW—unprocessed spectral data, SNV—standard normal variate, SG1—Savitzky–Golay second order derivative first order polynomial with 15 smoothing points, SG2—Savitzky–Golay second order derivative second order polynomial with 15 smoothing points, MSC—multiplicative scatter correction, RMSEP—root mean square error of prediction, RSEP—relative standard error of prediction, RPD—ratio of prediction, RER—range error ratio.

**Table 4:** Test statistics of the optimally developed partial least squares regression (PLSR) models to quantify adulteration of milk powder with corn starch and wheat flour (*n* = 70).

Math Pre-Treatment	Parameter	Model Validation Stage	RMSEP [%*w*/*w*]	RSEP [%]	RPD	RER	Factor
SG2 + SNV	Corn starch	Calibration	0.96	3.5	14.6	39.2	1
		Cross-validation	1.04	3.8	13.4	42.8	1
		Test	0.76	3.0	12.0	33.4	1
MSC + SG1	Wheat flour	Calibration	0.90	3.3	15.6	47.7	1
		Cross-validation	0.98	3.6	14.2	43.7	1
		Test	0.77	3.0	11.6	35.6	1

SNV—standard normal variate, SG1—Savitzky–Golay second order derivative first order polynomial with 15 smoothing points, SG2—Savitzky–Golay second order derivative second order polynomial with 15 smoothing points, MSC—multiplicative scatter correction, RMSEP—root mean square error of prediction, RSEP—relative standard error of prediction, RPD—ratio of prediction, RER—range error ratio.

## Data Availability

Data are available upon request from the corresponding authors.

## Supplementary Materials

The following supporting information can be downloaded at: https://www.mdpi.com/article/10.3390/s26041304/s1, Figure S1: Space-filling validation design utilized for the development of the PLSR models with calibration (blue squares) and test (orange squares) samples ordered from lowest to highest adulterant level; Table S1: Percentage distribution of calibration and test samples for the development of the PLSR models; Table S2: Thirteen matrices with all Raman spectra used for developing the PLSR models with different data preprocessing (DP) methods; Table S3: Optimal latent variable (LV) per partial least squares regression (PLSR) chemometrics model for the independent corn starch and milk powder (CS-MP) binary mixtures; Table S4: Optimal latent variable (LV) per partial least squares regression (PLSR) chemometrics model for the independent wheat flour and milk powder (WF-MP) binary mixtures. Table S5. Performance results for the PLSR model with optimal parameters (MSC) and the model with the optimal parameters using only one latent variable (MSC + SG1) for the independent wheat flour and milk powder (WF-MP) binary mixtures.

## Abbreviations

The following abbreviations are used in this manuscript:

%*w*/*w*	Weight-weight percentage
CAL	Calibration set
CS	Corn starch
CV	Cross-validation
FTIR	Fourier Transform infrared
GenAI	Generative artificial intelligence
MIR	Mid-infrared
MP	Milk powder
MSC	Multiplicative scatter correction
N	Total samples
NIPALS	Non-iterative partial least squares
NIR	Near-infrared
PCA	Principal component analysis
PLSR	Partial-least squares regression
RAW	Unprocessed spectral data
RER	Range error ratio
RMSEP	Root mean square error of prediction
RPD	Ratio of prediction
RS	Raman spectroscopy
RSEP	Relative standard error of prediction
SERS	Surface-enhanced Raman spectroscopy
SG1	Savitzky–Golay first derivative
SG2	Savitzky–Golay second derivative
SNV	Standard normal variate
TEST	External testing set
WF	Wheat flour
X	Predictor variables (Raman spectra)
Y	Response variable (adulterant concentration)
