# Supplementary material for: Detection and Quantification of Corn Starch and Wheat Flour as Adulterants in Milk Powder by Raman Spectroscopy Coupled with Chemometric Routines
Source: Sensors (Basel). 2026 Feb 18;26(4):1304. doi: 10.3390/s26041304 (PMC12944320; doi:10.3390/s26041304)
Supplement: Supplementary file 1 [file sensors-26-01304-s001.zip › sensors-4125761-supplementary.pdf]

sensors

MDPI

Supplementary

Detection and quantification of corn starch and wheat flour as adulterants in milk powder by Raman spectroscopy coupled with chemometric routines

Edwin R. Caballero-Agosto [1], Louang Cruz-Dorta [1], Samuel P. Hernandez-Rivera [2], Leonardo C. Pacheco-Londoño [2,3], and Ricardo Infante-Castillo [1,*]

[1] Department of Physics-Chemistry, University of Puerto Rico-Arecibo, Arecibo, PR 00614, USA
[2] Center for Chemical Sensors (CCS), Chemical Imaging and Surface Analysis Center (CISAC), Department of Chemistry, University of Puerto Rico-Mayagüez, Mayagüez, PR 00681, USA
[3] Faculty of Basic and Biomedical Sciences-Barranquilla, Simon Bolivar University, Barranquilla 080002, Colombia
* Correspondence: ricardo.infante1@upr.edu; (787) 470-9273

Supplementary Materials

Sample Heterogeneity Analysis

To estimate the heterogeneity of the mixtures, the six replicate spectra acquired at different points of each sample were evaluated using three prominent peaks: peak 1 (323-380 cm^{-1}), peak 2 (454-540 cm^{-1}), and peak 3 (1420-1500 cm^{-1}). Relative standard deviation (RSD) values were calculated for each band across replicates. This method was used to capture local compositional variability while minimizing noise-dominated spectral regions. After calculating the average and standard deviation for each peak and concentration, the overall average and standard deviation were calculated across the three peaks and concentrations to obtain an overall RSD value per adulterant. Results show an overall RSD of 12.0% for MP-CS and 7.4% for MP-WF.

External Validation Design

The following Figure S1 and Table S1 illustrate the external validation design utilized for the development of the partial least squares regression (PLSR) models for the individual binary mixtures of cornstarch (CS) and milk powder (MP), and wheat flour (WF) and MP, consisting of 26 calibration (CAL) samples and 9 test (TEST) samples.

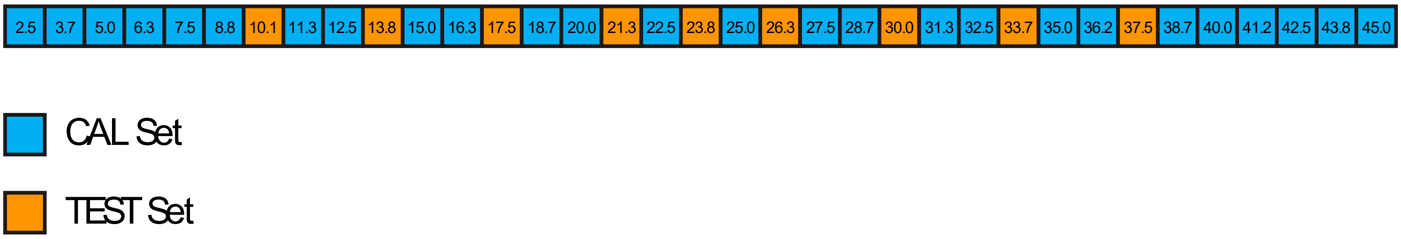

Figure S1. Space-filling validation design utilized for the development of the PLSR models with calibration (blue squares) and test (orange squares) samples ordered from lowest to highest adulterant level.

 https://doi.org/10.3390/s26041304

Table S1. Percentage distribution of calibration and test samples for the development of the PLSR models.

Set	Weight-Weight Percentage (%w/w)												
Calibration	2.5	3.7	5.0	6.3	7.5	8.8	11.3	12.5	15.0	16.3	18.7	20.0	22.5
	25.0	27.5	28.7	31.3	32.5	35.0	36.2	38.7	40.0	41.2	42.5	43.8	45.0
Validation	10.1	13.8	17.5	21.3	23.8	26.3	30.0	33.7	37.5				

Partial Least Squares Regression (PLSR) Models Discussion

The PLSR models were developed using a total of six latent variables (LVs) individually for a total of 13 models consisting on the use of unprocessed (RAW) Raman spectral data as well as different combinations of scattering correction data preprocessing methods (Standard Normal Variate, SNV; Multiplicative Scatter Correction, MSC) and Savitzky-Golay second polynomial derivatives (First derivative order, SG1; second derivative order, SG2) with 15 smoothing points. Table S2 shows the thirteen models developed.

Table S2. Thirteen matrices with all Raman spectra used for developing the PLSR models with different data preprocessing (DP) methods.

Model	M01	M02	M03	M04	M05	M06	M07	M08	M09	M10	M11	M12	M13
DP	RAW	MSC	SNV	SG1	SG2	MSC+SG1	MSC+SG2	SNV+SG1	SNV+SG2	SG1+MSC	SG2+MSC	SG1+SNV	SG2+SNV

Table S3 shows the optimal LV for all 13 PLSR models for CS-MP, and Table S4 shows the optimal LV for WF-MP. Table S3 shows that for 69% of the models, optimal results were obtained using only one LV, with RAW and SG1 having the highest number of LVs; M13 (SG2+SNV) had the lowest RMSEP and RSEP, as well as higher RPD and RER. Table S4 shows a different story for WF-MP than CS-MP regarding LVs.

Table S3. Optimal latent variable (LV) per partial least squares regression (PLSR) chemometrics model for the independent corn starch and milk powder (CS-MP) binary mixtures.

Model	DP	LV	RMSEP	RSEP (%)	RPD	RER
M01	RAW	6	1.21	4.8	7.3	22.1
M02	MSC	1	0.96	3.8	9.6	26.1
M03	SNV	4	0.94	3.7	9.4	28.7
M04	SG1	6	1.06	4.2	8.4	24.5
M05	SG2	5	1.12	4.4	7.9	23.8
M06	MSC+SG1	1	0.79	3.1	11.2	31.4
M07	MSC+SG2	1	0.76	3.0	11.9	33.0
M08	SNV+SG1	1	0.80	3.2	11.2	31.7
M09	SNV+SG2	1	0.76	3.0	12.0	33.1
M10	SG1+MSC	1	0.83	3.3	10.6	31.3

M11	SG2+MSC	1	0.76	3.0	11.8	33.1
M12	SG1+SNV	1	0.81	3.2	11.1	32.5
M13	SG2+SNV	1	0.75	3.0	12.1	33.6

In Table S4, the average number of LVs that provided optimal results was 4, while the lowest was 2 for model M07 (MSC+SG2). When analyzing the results of models with only one LV, the optimal model was M06 (MCS+SG2) with 0.77 %w/w RMSEP, 3.0% RSEP, 11.6 RPD, and 35.6 RER, not M07 (MSC+SG2) with 0.80 %w/w RMSEP, 3.2% RSEP, 11.3 RPD, and 34.5 RER. When analyzing the performance of model M06 with the models with optimal LVs, M06 has lower RMSEP M04, M05, M10, and M11, lower RSEP than M04, M05, M10, M11, and M13, higher RPD than M05, M10, and M11, and higher RER than M04, M05, M09, and M10. The performance of model M06 with one LV was better than 30% of the models with optimal LV, with differences of 0.06% w/w RMSEP, 0.2% RSEP, 1.2 RPD, and 2.5 RER compared to M07 with two LVs; a difference below 10% for all parameters. The difference between M06 with one LV and M07 with two LVs is not negligible. However, compared with how the first component in the PCA scores of Figure 3-b discriminates between adulterant percentages, the difference of -2250 to 2250 for PC2, compared with -4200 to 2140 for 29% and 95% explained variance, can justify the reduction in complexity from two LVs to one. This analysis compares the model with the lowest LVs from Table S4 (M07) with the optimal model of Table S4 (M02; MSC) using 5 LVs. We see an RMSEP of 0.57 %w/w, RSEP of 2.2%, RPD of 16.4, and RER of 47.6. Table S5 illustrates that when comparing the PLSR model M06 with one LV to M02 with 5 LVs, we see an average difference of 31%, which is not negligible; however, the added complexity of four additional LVs is not desired when one of the criteria of the PLSR model is to have the least amount of complexity (i.e., a parsimonious model).

Table S4. Optimal latent variable (LV) per partial least squares regression (PLSR) chemometrics model for the independent wheat flour and milk powder (WF-MP) binary mixtures.

Model	DP	LV	RMSEP	RSEP (%)	RPD	RER
M01	RAW	4	0.71	2.8	13.1	37.2
M02	MSC	5	0.57	2.2	16.4	47.6
M03	SNV	6	0.58	2.3	16.0	45.2
M04	SG1	5	0.78	3.1	11.6	33.6
M05	SG2	3	0.85	3.4	10.6	29.4
M06	MSC+SG1	5	0.71	2.8	12.8	38.1
M07	MSC+SG2	2	0.72	2.8	12.7	35.9
M08	SNV+SG1	3	0.71	2.8	12.9	36.6
M09	SNV+SG2	4	0.73	2.9	12.7	35.2
M10	SG1+MSC	4	0.93	3.7	9.6	31.4
M11	SG2+MSC	4	0.88	3.5	10.5	36.4
M12	SG1+SNV	4	0.71	2.8	12.9	35.9
M13	SG2+SNV	4	0.77	3.0	12.2	36.1

Table S5. Performance results for the PLSR model with optimal parameters (MSC) and the model with the optimal parameters using only one latent variable (MSC+SG1) for the independent wheat flour and milk powder (WF-MP) binary mixtures.

Model	DP	LV	RMSEP	RSEP (%)	RPD	RER
M02	MSC	5	0.57	2.2	16.4	47.6
M06	MSC+SG1	1	0.77	3.0	11.6	35.6

The samples prepared in this study will not fully reflect the complexity of the samples to be analyzed in their designated areas of interest. These samples may vary in the quality of their components, sample humidity, and environmental conditions, and may contain other added components. Additionally, other factors include the proper acquisition angle and the spectrometer-to-sample distance when used by non-experts in the field. All these factors will contribute to unwanted variations or artifacts that will change the acquired spectra. The more LVs a chemometric model contains, the greater its dependence on the specific structure of its calibration samples. This dependency can increase the likelihood that the model incorrectly quantifies or discriminates unknown samples, in part because spectral artifacts between the unknown and calibration samples are significantly different. This difference can lead to incorrect predictions even when the chemical information is present, resulting in lower model robustness. Therefore, using low amounts of LVs can result in chemometric models that are less robust to factors not considered in standardized laboratory practices, which are not the practices expected for the impacted group of users for whom this study is targeted.
